# A Comprehensive HLA-DR4 MHC Class II Tetramer Platform for the Detection and Functional Validation of Post-Translational Modification Neoantigens

**DOI:** 10.3390/ijms27135660

**Published:** 2026-06-23

**Authors:** Henghui Li, Jingyao Li, Ying Wang, Hongyan Ma, Fen Tang, Liang Chen

**Affiliations:** School of Medicine, Shanghai University, Shanghai 200444, China

**Keywords:** post-translational modification (PTM) neoantigens, major histocompatibility complex (MHC) class II tetramer, HLA-DR4, autoimmune diseases (AUIDs), fluorescence polarization (FP), high-throughput screening, T-cell receptor (TCR) recognition, antigen-specific CD4^+^ T cells

## Abstract

Post-translational modification (PTM) neoantigens have emerged as key drivers of autoimmune inflammation. However, standardized protocols for MHC Class II tetramer preparation for the detection of such antigen-specific T cells remain limited, hindering the broader application of this important discovery. This study systematically engineered an HLA-DR4 (HLA-DRB1*04:02 and HLA-DRA*01:01) tetramer platform based on carboxyethyl-modified neoantigen ITGA2B peptide (ITG-CE), a PTM associated with autoimmune diseases (AUIDs) such as Ankylosing Spondylitis (AS). The platform provides a major histocompatibility complex (MHC) Class II tetramer associated with the PTM neoantigen and integrates modular protein construct, a controllable PTM peptide exchange strategy, and a specific T cell receptor (TCR) validation model. It can be employed to investigate PTM neoantigen presentation and CD4^+^ T cell auto-reactivity, providing extensive application value for future research into the mechanisms of PTM-induced AUIDs and immune monitoring.

## 1. Introduction

Autoimmune diseases (AUIDs) exhibit a robust genetic predisposition with major histocompatibility complex (MHC) genes, a pattern confirmed in multiple conditions including Rheumatoid Arthritis (RA), Type 1 Diabetes (T1D), Inflammatory Bowel Diseases (IBD), and Systemic Lupus Erythematosus (SLE) [1,2,3]. In humans, the high polymorphism of MHC molecules, known as human leukocyte antigens (HLA), constitutes one of the most significant genetic susceptibility factors for multiple AUIDs.

Within the MHC class I repertoire, HLA-B27 displays the most pronounced linkage to Ankylosing Spondylitis (AS), with a positive rate of 80–90% among affected patients [4,5,6,7]. This represents one of the strongest HLA-disease couples identified to date among known common polygenic disorders [8]. Furthermore, HLA-C*06:02 constitutes a primary genetic risk factor for plaque psoriasis [9,10,11]. In contrast, MHC Class II molecules exhibit broader associations with AUIDs and more clearly defined mechanisms. Taking RA as an example, the DR4 allele represents a well-established genetic susceptibility factor for RA. Mechanistically, the shared epitope hypothesis attributes this risk to a motif at positions 70–74 of the DRβ chain, a feature common to DR4 alleles (HLA-DRB1*04:01, HLA-DRB1*04:04, HLA-DRB1*04:05) and HLA-DRB1*01:01 [12,13]. This structural feature alters the conformation of the antigen-binding pocket, thereby influencing antigen peptide binding and T cell activation [14,15]. In T1D, the genetic effects of MHC Class II molecules are equally significant. The “DR3” and “DR4” haplotypes carried by heterozygotes in individuals bear the higher disease risk than homozygous individuals [16,17], with the 57th non-Asp in the DQβ chain molecule highly correlated with the risk of diabetes [18]. However, other studies indicate that DQB1*06:02, carrying Asp57, confers significant protective effects [19]. In multiple sclerosis (MS) and SLE, the DR15 subtype (HLA-DRB1*15:01) is closely associated with the development of diseases [20,21,22,23]. Moreover, HLA-DQ2 is recognized as a major genetic susceptibility factor for celiac disease [24,25]. Notably, MHC alleles exhibit marked variation across populations. For instance, Caucasians and Asian populations harbor distinct dominant genotypes [26,27]. Such disparities in population genetic structure not only influence disease epidemiology but also complicate studies investigating mechanisms with cross-population universality. Currently, although extensive genetic studies have revealed association patterns of disease-related MHC molecules, the specific antigen presentation profiles and their function in pathogenesis remain incompletely elucidated [28,29]. In this context, the development of peptide-MHC (pMHC) tetramers provides a crucial technical tool for directly identifying antigen-specific T cells and deciphering disease-associated antigen presentation mechanisms.

In the identification of antigens associated with AUIDs, the discovery of autoantigenic presents greater challenges than that of tumor antigens. This complexity arises from the multiple pathways involved in autoantigen production. Beyond epitopes derived from classical proteolytic cleavage pathways [30], peptides generated through post-translational modifications (PTMs) can disrupt immune tolerance by altering the chemical structure and immunogenicity of endogenous proteins [31,32,33]. Accumulating evidence identifies aberrant protein citrullination as a key contributor to the initiation and advancement of RA pathology [34,35]. Despite growing recognition of the pivotal role played by PTM derived neoantigens in the initiation and maintenance of AUIDs, tools capable of directly detecting these epitope specific CD4^+^ T cells remain severely limited. Concurrently, the in vitro acquisition of PTM neoantigens faces numerous instability factors, further constraining systematic investigations into their pathogenic mechanisms. Our previous research identified a PTM epitope carboxyethyl-modified neoantigen ITGA2B peptide (ITG-CE), which is generated when the 96th-position cysteine of CD41 undergoes carboxyethyl modification. This epitope is presented by theHLA-DR4 (HLA-DRB1*04:02 and HLA-DRA*01:01) allele and drives disease progression in AS, whilst showing associations with RA and SLE [36]. This discovery establishes PTM neoantigens as disease-relevant targets, simultaneously highlighting the urgent need for robust platforms capable of reliably detecting PTM-specific T cells.

MHC tetramers serve as vital tools for tracking antigen-specific T cells. However, current MHC Class II tetramer platforms still exhibit critical gaps in assessing the processing, presentation, and T cell recognition of PTM neoantigens [37,38]. The effective application of MHC tetramer technology to PTM neoantigen research necessitates rigorous control over monomer quality, peptide loading fidelity, and tetramer stability. Factors that may vary significantly depend on the expression systems and assembly strategies used. Existing workflows provide basic conceptual strategies but lack a unified system for evaluating PTM peptide affinity for DR4 allele, structural compatibility, and T cell receptor (TCR) specificity within a single experimental framework [39,40]. These technical bottlenecks constrain in-depth studies of PTM-specific autoreactive T cells, necessitating the establishment of standardized MHC Class II tetramer workflows optimized for PTM neoantigens.

To address this gap, this study utilized ITG-CE as a model to establish an HLA-DR4 tetramer workflow optimized for PTM neoantigens. First, we designed a modular in vitro expression motif of HLA-DR4 to evaluate construct integrity, tag accessibility, and overall monomer behavior. Building upon this foundation, we provide standardized fluorescence polarization (FP) competitive binding assays for quantitatively assessing interactions between HLA-DR4 and cysteine carboxyethylation-modified peptides, demonstrating excellent adaptability to PTM neoantigens. Considering that PTM peptide-loaded MHC Class II tetramers may face additional challenges in stability and functional activity, we systematically optimized parameters related to tetramer assembly and T cell staining. We established a validation protocol for cysteine carboxyethylation-modified epitopes based on TCR recognition. Through the above integration strategy, we have built a comprehensive HLA-DR4 tetramer platform that not only supports in vitro functional characterization of PTM neoantigen pMHC complexes but also enables batch-to-batch consistency in quality control and validation.

The innovation of this study lies in establishing a systematic and scalable in vitro validation platform for PTM epitopes, directly linking PTM biological characteristics with allele-specific autoimmune risk, which provides a novel technical pathway for elucidating the pathogenesis of AUIDs. Furthermore, the platform can be extended to discover neoantigens from other PTM types and investigate their HLA-allele presentation mechanisms, thereby laying a reliable methodological foundation for broader MHC Class II neoantigen research. In the long term, the standardized technology system established by this platform is expected to provide stable and reproducible technical support for elucidating the underlying mechanisms of clinically relevant AUIDs and developing novel therapeutic strategies.

## 2. Results

### 2.1. Design and Characterization of HLA-DR4 Protein Complexes

**Figure 1:**
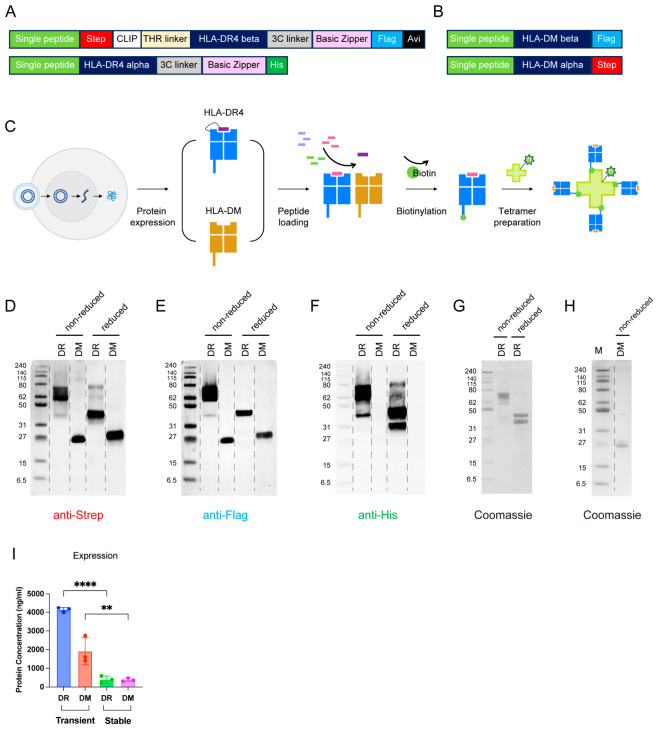
HLA-DR4 protein construct and expression system design. (**A**) The design of the engineered HLA-DR4 protein construct. (**B**) The design of the engineered HLA-DM protein construct. (**C**) The workflow for recombinant expression of HLA-DR4 and HLA-DM protein, followed by antigen peptide loading and HLA-DR4 tetramer preparation. (**D**–**F**) The HLA-DR4 and HLA-DM were verified by Western blot under reducing and non-reducing conditions. Western blot with anti-Strep antibody (**D**), anti-Flag antibody (**E**) and anti-His antibody (**F**). (**G**) Coomassie blue staining of purified HLA-DR4 monomer protein under reduced or non-reduced conditions. (**H**) Coomassie blue staining of purified HLA-DM monomer protein under non-reduced conditions. (**I**) Quantification of HLA-DR4 and HLA-DM protein expression yield (*n* = 3). Data are presented as mean ± SD. Differences between groups were analyzed by one-way ANOVA with Tukey’s multiple-comparisons test using GraphPad Prism software (version 10.0). ** *p* < 0.01; **** *p* < 0.0001.

To establish a platform compatible with PTM epitope presentation, we designed a modular and scalable in vitro expression construct for HLA-DR4 protein (Figure 1A). The construct adopts a multi-functional modules strategy with clearly defined functions: affinity tags facilitate protein enrichment and purification; removable placeholder peptides (CLIP peptides) facilitate high-throughput peptide exchange; enzymatic biotinylation sites enable downstream tetrameric assembly; and a dimerization zipper structure ensures proper folding and integrity verification of heterodimers. The integration of the above modules enables this construct to achieve comprehensive coverage from quality control to functional validation within a single expression system.

In the design strategy for the construct, the conserved region sequences of HLA-DR4 were retrieved from the International ImMunoGeneTics information system (IMGT) database, with modular functional tags and heterodimerizing zipper structures integrated upon this foundation. Specifically, the Strep Tag verifies binding and dissociation of the CLIP peptide, while the Flag Tag and His Tag were used to confirm the molecular weight and correct folding of the HLA-DR4 β and HLA-DR4 α chains, respectively. Notably, due to the absence of transmembrane domains, HLA-DR α and β chains expressed in vitro tend to exist as multimers [41]. To address this, we inserted basic leucine zippers and acidic leucine zippers at the C-terminal of the β and α chains, respectively, to promote correct monomeric folding while effectively suppressing oligomer formation. Conventional approaches for evaluating HLA-DR4 structural integrity and peptide-binding properties, including mass spectrometry, Size Exclusion Chromatography with Multi-Angle Light Scattering (SEC-MALS), surface plasmon resonance (SPR), or Bio-Layer Interferometry (BLI), and ELISA, generally require specialized instrumentation, substantial sample consumption, or additional antibody reagents [42,43,44]. In contrast, the present construct incorporates multiple functional tags, enabling straightforward assessment of protein expression, heterodimer assembly, and peptide-binding status using routine molecular biology techniques. This construct offers a lower technical threshold and broader laboratory applicability for evaluating HLA-DR4 protein expression quality, dimer integrity, and folding status. Furthermore, this scheme is compatible with multiple HLA-DR alleles including DR1 (DRA, DRB1*01:01), DR15 (DRA, DRB1*15:01) and DR4 allele (DRA, DRB1*04:01), conferring robust versatility and reproducibility to the platform.

For the modular design of the chaperonin HLA-DM [45], its construct similarly relies on conserved regions sequence from the IMGT database, complemented by functional tags: Strep Tag and Flag Tag were employed to assess the α and β chains of HLA-DM respectively (Figure 1A). Given that HLA-DM must be removed from the system after assisting HLA-DR in high-affinity antigen peptide loading (Figure 1B), the His Tag was specifically engineered onto the HLA-DR molecule rather than the HLA-DM protein, enabling selective separation and purification of the two components. This design reflects the modular core principle of the construct system, where each functional tag can be flexibly replaced or expanded according to laboratory conditions and research requirements, thereby maximizing the adaptability and operational convenience of the platform.

### 2.2. In Vitro Preparation and Quality Assessment of HLA-DR4 Complexes

As illustrated, the figure shows the tags’ accessibility and structural integrity of HLA-DR4 monomer protein (Figure 1C,D). Due to the presence of the leucine zipper, the α and β chains of the HLA-DR4 molecule correctly fold into heterodimeric forms during expression. Under non-reducing conditions, HLA-DR4 maintains its dimeric structure with a molecular weight of 72 kDa. However, under reducing conditions, the leucine zipper collapses, causing HLA-DR4 to adopt α and β single-chain forms with molecular weights of 32 kDa and 40 kDa, respectively. Coomassie results confirm that the molecular weights of HLA-DR4 protein under reducing and non-reducing conditions align with expectations (Figure 1G). Furthermore, Western blot analysis showed that the Strep Tag and Flag Tag labeled the β chain, while the His Tag labeled the α chain (Figure 1D–F). These tags were present in both the dimeric and monomeric forms with correct sizes, corresponding to the Coomassie results. This indicates that the HLA-DR4 dimer produced possesses the correct structure.

In contrast, the chaperonin HLA-DM protein lacks a leucine zipper and thus exists as a single chain under non-reducing conditions (Figure 1H). The α and β chains of HLA-DM protein exhibit molecular weights of 27.4 kDa and 26.7 kDa, respectively. Western blot analysis confirmed the presence of the Strep Tag and Flag Tag on the HLA-DM protein, corresponding to its α and β chains, respectively, while the His Tag signal was entirely absent as it was uniquely engineered onto the HLA-DR4 molecule (Figure 1D–F). These results further confirm the structural integrity and purity of the produced HLA-DM complexes.

To facilitate stable HLA-DR4 monomer production, we attempted stable cell line establishment and provided relative yield estimates. Compared to the transient expression system, the protein yield obtained from the stabilized cell line was significantly insufficient (Figure 1D). Therefore, stable expression engineered from the ExpiSf9 cell line is not recommended.

### 2.3. Design of Intermediate Peptides for PTM Neoantigen Affinity Evaluation

**Figure 2:**
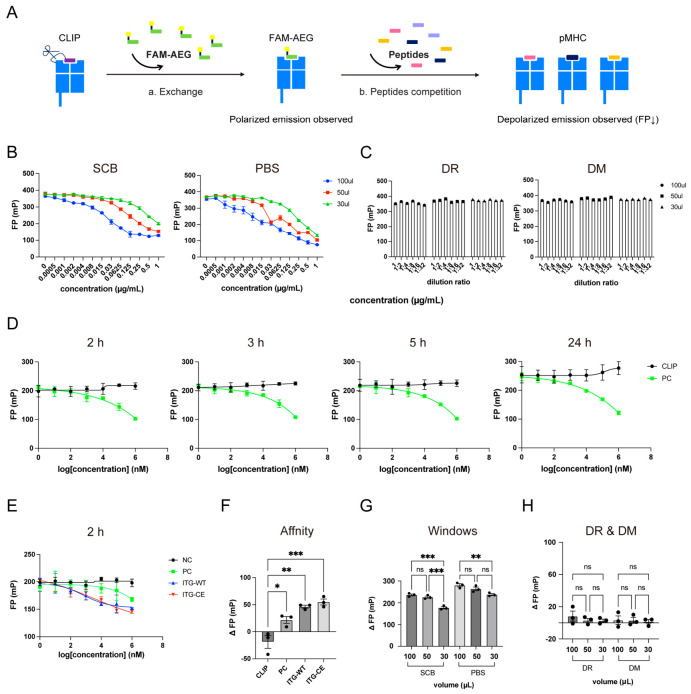
PTM neoantigen peptide binds to HLA-DR4 monomer protein with high affinity. (**A**) Schematic of the optimized FP strategy for high-throughput antigen peptide screening. (**B**) Concentration gradient curves of FAM-AEG in different buffers (*n* = 3). (**C**) Background interference signals and FP values of additional composition (HLA-DR4 and HLA-DM) (*n* = 1). (**D**) FP results of different incubation time for PC peptide competitive exchange (*n* = 3). (**E**) Assessment of the PTM ITG-CE peptide and other unrelated peptides’ affinity for HLA-DR4 (*n* = 3). (**F**) Quantification analysis of the ITG-CE peptide and other unrelated peptides’ affinity for HLA-DR4 (*n* = 3). $∆FP=FP\left(experimental\;group\right)-FP(blank\;group)$. Experimental group, concentrations of 1 mM peptide. Blank group, buffer only. (**G**) Quantification analysis of the background effects from SCB and PBS buffer (*n* = 3). $∆FP=FP\left(experimental\;group\right)-FP(blank\;group)$. Experimental group, buffer dissolved different concentrations of FAM-AEG peptide. Blank group, buffer only. (**H**) Quantification analysis of the background interference of HLA-DR and HLA-DM components (*n* = 3). Data are presented as mean ± SEM. Statistical significance was determined using one-way ANOVA with Tukey’s multiple-comparisons test. ns, not significant; * *p* < 0.05; ** *p* < 0.01; *** *p* < 0.001.

We employed a FP approach to evaluate interactions between HLA-DR4 and the PTM epitope ITG-CE. Different from conventional FP experiments, which typically involve FAM or Alexa Fluor 488 (AF488) labeling on each screened peptide [46], here we designed a fluorescently labeled intermediate peptide (FAM-AEG) to serve as a baseline for assessing the affinity of candidate antigen peptides towards the HLA-DR4 molecule (Figure 2A). By tracking changes in the polarized light signal during binding and dissociation of the intermediate peptide with HLA-DR4 dimers, we screened for high-affinity candidate antigen peptides capable of competing against FAM-AEG from HLA-DR4. The EBNA1482-496 intermediate peptide was screened from the database (https://www.proimmune.com/). Its affinity for HLA-DR4 was lower than that for the PC peptide but higher than that for the ITG-WT peptide.

In this study, to rigorously control interference signals, we systematically analyzed the background interference factors during peptide screening, including buffer composition, reaction volume, and incubation pH value conditions. First, FAM-AEG was gradient diluted using different reaction buffers (PBS or SCB), with FP values recorded across varying volumes (Figure 2B,G). The results corroborated that different buffers did not affect the integrity of the ΔFP window, meaning initial and final values remained consistent. However, the S-curve was more pronounced at a 100 μL reaction volume of SCB, compared to PBS, potentially due to buffer composition and the degree of liquid surface concavity affecting FP value readings. Concurrently, FP values of HLA-DM and HLA-DR4 were found not to be sensitive to buffer type (Figure 2C,H). Therefore, we ultimately selected a 100 μL reaction volume, SCB, and HLA-DM as the catalyst for subsequent high-throughput peptide screening.

Subsequently, we utilized the PC peptide to evaluate the incubation time for the peptide exchange reaction. As anticipated, the PC peptide competitively binds to the intermediate product FAM-AEG/DR4 in a concentration-dependent manner, displacing FAM-AEG (Figure 2D). Moreover, the results from a 2 h incubation were consistent with 3 h and 5 h, indicating that 2 h provided sufficient reaction time for the competitive displacement process. Conversely, a 24 h incubation led to a marked shift in the curve due to evaporation (Figure 2D). Under these conditions, we assessed the affinity of the ITG-CE with HLA-DR4 within 2 h, consistent with its expected role as an autoimmune neoepitope (Figure 2E). Here, the PC peptide, CLIP peptide, and ITG-WT peptide served as controls. The ITG-CE peptide exhibited measurable binding behavior within 2 h (Figure 2F). Interestingly, the ITG-WT peptide also displaced FAM-AEG (Figure 2E,F), suggesting ITG-WT may possess binding capacity but fail to generate detectable tetramer staining under the tested conditions. This indicates that the wild-type sequence retains baseline binding affinity for the HLA-DR4 groove. However, as demonstrated in subsequent flow cytometry, this baseline binding does not translate into T-cell recognition, confirming that the post-translational carboxyethylation is structural-critical for specific TCR engagement and subsequent auto-reactivity.

### 2.4. HLA-DR4 Tetramer Assembly and Structural Validation

**Figure 3:**
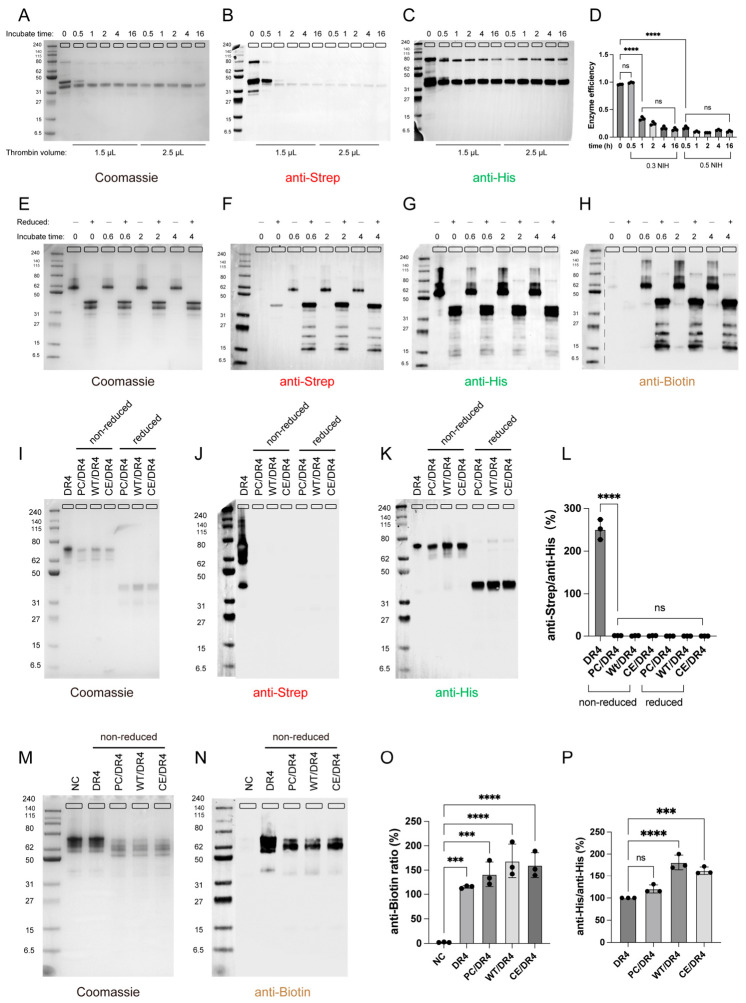
Peptide-loaded HLA-DR4 monomer preparation for tetramer assembly. (**A**–**C**) Removal of CLIP placeholder peptides. Coomassie blue staining of HLA-DR4 complexes incubated with thrombin (**A**), Western blot with anti-Strep antibody (**B**), and anti-His antibody (**C**). (**D**) Quantification analysis of enzymatic cleavage efficiency for HLA-DR4 complexes (*n* = 3). (**E**–**H**) Time required to complete the biotinylation of HLA-DR4. Coomassie blue staining of HLA-DR4 complexes incubated with biotin ligase (**E**). Western blot with anti-Strep antibody (**F**), anti-His antibody (**G**), and anti-Biotin antibody (**H**). (**I**–**K**) His-Tag affinity purification of HLA-DR4 pMHCs (PC/DR4, WT/DR4, CE/DR4) following peptide exchange. Coomassie blue staining of purified pMHCs (**I**). Western blot with anti-Strep antibody (**G**) and anti-His antibody (**K**). (**L**) Quantification analysis of CLIP removed (*n* = 3). (**M**,**N**) Biotinylation verification of the HLA-DR4 pMHCs (DR4, PC/DR4, WT/DR4, CE/DR4). Coomassie blue staining of biotinylation pMHCs (**M**), and Western blot with anti-Biotin antibody (**N**). (**O**) Quantification analysis of biotinylation for HLA-DR4 complexes (*n* = 3). (**P**) Quantification analysis of purification for HLA-DR4 pMHCs (*n* = 3). Data are presented as mean ± SD. Statistical significance was determined using one-way ANOVA with Dunnett’s multiple-comparisons test and Tukey’s multiple-comparisons test. ns, not significant; *** *p* < 0.001; and **** *p* < 0.0001.

To prepare pMHC monomers for tetramer assembly, we first validated the removal of the CLIP occupant peptide and the biotinylation of the monomer. HLA-DR4 monomer linked CLIP (DR4) and processed without thrombin (THR) served as a negative control. Coomassie staining revealed that the HLA-DR4 dimer dissociated into β (40 kDa) and α (32 kDa) chains under reducing conditions. Following complete enzymatic digestion, the β chain molecular weight decreased by approximately 7 kDa. Consequently, with increasing incubation time of thrombin, the band around 40 kDa disappeared, and the molecular weight of the β chain gradually matched that of the α chain, which was consistent with expectations (Figure 3A). Strep Tag was used to characterize the CLIP peptide. Upon completion of enzymatic cleavage, the CLIP peptide was removed, and the Strep Tag disappeared (Figure 3B). Removal of the CLIP peptide required 1–2 h with 0.3 NIH thrombin, whereas 0.5 NIH thrombin completed the process, taking only 0.5 h (Figure 3A–D). Throughout this procedure, the α chain (His-tagged) remained fully intact, with comparable protein loading across all lanes (Figure 3C).

During the biotinylation process, Western blot results of anti-biotin antibodies showed that 100% biotinylation could be completed after 40 min of incubation, compared to the control group (Figure 3H), with consistent outcomes for both reduced and non-reduced samples. Coomassie staining indicated intact monomeric structure, with non-reduced samples existing as α and β complexes, while reduced samples showed dissociation of α and β into single chains (Figure 3E). Strep Tag and His Tag Western blot results further confirmed that the biotinylation process preserved the HLA-DR4 monomer structure (Figure 3F,G).

Based on the above conditions, we prepared peptide loaded HLA-DR4 pMHC complexes. Through peptide exchange and purification, we obtained PC peptide-loaded HLA-DR4 pMHC (PC/DR4), ITG-WT peptide-loaded HLA-DR4 pMHC (WT/DR4), and ITG-CE peptide-loaded HLA-DR4 pMHC (CE/DR4). Untreated DR4 served as control (Figure 3I). Results showed that PC/DR4, WT/DR4, and CE/DR4 underwent complete cleavage with no Strep Tag residual (Figure 3J,L), compared to DR4. PC/DR4, WT/DR4, and CE/DR4 successfully yielded high-purity products following His-Tag affinity purification (Figure 3K,P). Furthermore, biotinylation was successfully completed on PC/DR4, WT/DR4, and CE/DR4 under both reducing and non-reducing conditions (Figure 3M–O).

**Figure 4:**
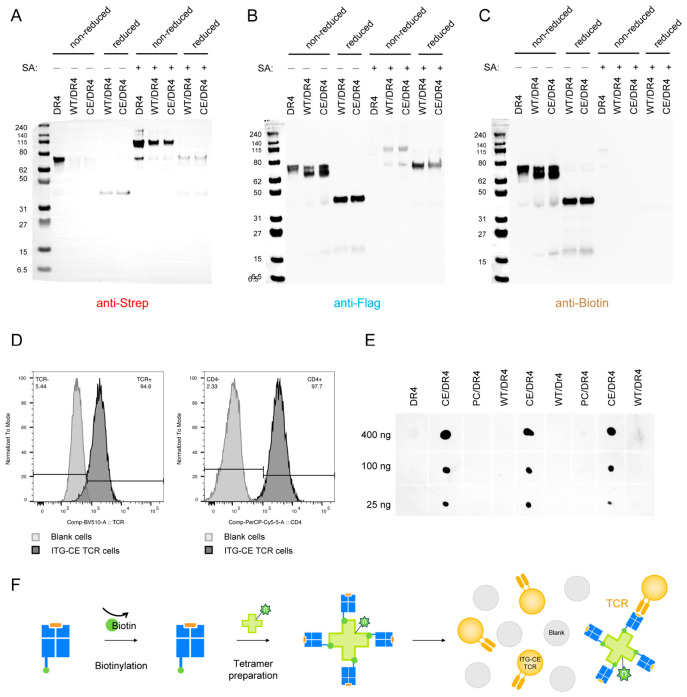
HLA-DR4 tetramers loaded with PTM peptides exhibit structural integrity. (**A**–**C**) Western blot of pMHC (DR4, WT/DR4, CE/DR4) and SA with anti-Strep antibody (**A**), anti-Flag antibody (**B**) and anti-Biotin antibody (**C**). (**D**) Flow cytometry detection of ITG-CE specific TCR-expressing cell lines. (**E**) Dot blot confirming correct loading of the ITG-CE peptide onto HLA-DR4. (**F**) Quantification analysis of dot blot results (*n* = 3). Data are presented as mean ± SD. Statistical significance was determined using one-way ANOVA with Tukey’s multiple-comparisons test.

The Strep-tagged CLIP occupant peptide was completely removed. However, Strep Tag was detectable in the non-reduced sample after SA addition, likely because the anti-Strep antibody recognizes SA, while SA depolymerization under reduced conditions cannot be recognized by anti-Strep antibodies (Figure 4A). The Flag Tag demonstrated that the β chain, after CLIP removal, correctly folded into a dimeric form with the α chain, exhibiting the correct molecular weight (Figure 4B). However, since the C-terminus of the β chain binds to SA to form a tetramer, the Flag tag at the C-terminus of the β chain can be detected weakly by Western blotting once assembly is complete. As shown, all pMHC monomers were biotinylated (Figure 4C). The binding ability of biotinylated pMHC monomers to SA was validated by a pMHC:SA molar ratio of 1:2 to ensure all monomers bound SA with no free monomers present. Moreover, since excess SA binds surplus biotin molecules, no free biotin was present post-incubation (Figure 4C). These results are consistent with expectations.

### 2.5. HLA-DR4 Tetramers Specific Recognition of PTM Neoantigen

PTM neoantigen peptide loading was analyzed via dot blot assay. As anticipated, the anti-CE antibody exclusively bound modified peptides, exhibiting no affinity for wild-type peptides or unrelated antigenic peptides (Figure 4E). Furthermore, the specific recognition by anti-CE antibody demonstrated concentration-dependent sensitivity (Figure 4E). Collectively, the present data establish that side chain modifications introduced by PTM of antigenic peptides are structurally compatible with the HLA-DR4 peptide-binding pocket without disrupting the classical conformation.

**Figure 5:**
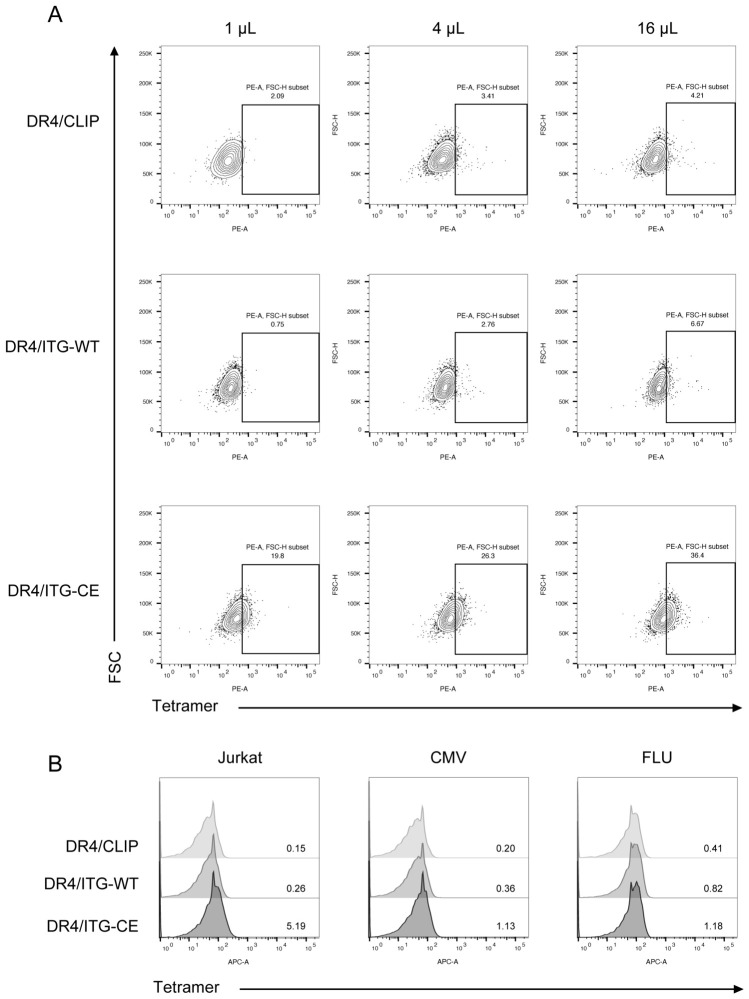
The HLA-DR4 tetramer loaded with PTM epitope exhibits a unique TCR recognition signal. (**A**) Flow cytometry for the HLA-DR4 tetramers (DR4/CLIP, DR4/ITG-WT, DR4/ITG-CE). (**B**) Flow cytometry for the DR4/ITG-CE tetramer of multiple unrelated TCR-expressing cells (wild-type Jurkat cells, CMV-J76, FLU-J76) to validate its biological functional specificity. The light gray, gray, and black closed curves represent samples stained with DR4/CLIP, DR4/ITG-WT, and DR4/ITG-CE tetramers, respectively.

We constructed TCR-expressing cell lines representative of both recognition associated and non-recognition associated TCRs to validate the biological function of the DR4/ITG-CE tetramer (Figure 4F). We first validated ITG-CE-specific TCR expression in CD4^+^ J76 cell lines, where ITG-CE-specific TCR-transduced CD4^+^ J76 cells (ITG-CE TCR cells) exhibited 94.6% TCR positive rate compared to un-transfected cells (Figure 4D). The neoantigen receptor (ITG-CE TCR) expressed by these cells carries direct biological and clinical relevance to autoimmune pathology, having been originally isolated and sequenced from primary CD4^+^ T cells of patients with AS following stimulation with modified peptide-pulsed mDCs [36]. Subsequent flow cytometric analysis corroborated that DR4/ITG-CE tetramer specifically recognized ITG-CE TCR cells, with ITG-CE TCR cells being exclusively positive for the DR4/ITG-CE tetramer and negative for the DR4/ITG-WT tetramer and DR4/CLIP tetramer, proving that tetramer recognition was PTM epitope-specific (Figure 5A). And the intensity of positive cells exhibited a dose-dependent relationship with the tetramer concentration. Concurrently, a stringent validation of DR4/ITG-CE tetramer specificity was performed using cell lines expressing unrelated TCRs (Figure 5B). The results substantiated that the DR4/ITG-CE tetramer did not respond to multiple cell lines bearing unrelated TCR backgrounds (Figure 5B), thereby ruling out non-specific binding [47]. In summary, the HLA-DR4 neoantigen tetramer selectively labeled ITG-CE-specific TCR cells. A rigorous and systematic framework for validating specific PTM neoantigens has been established.

### 2.6. Optimization and Validation of Tetramer Staining Performance

**Figure 6:**
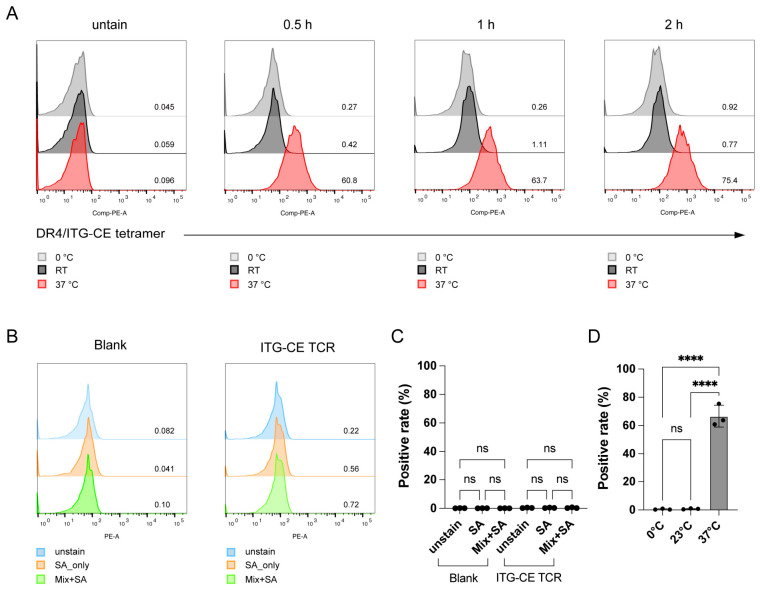
Rigorously validating DR4/ITG-CE tetramer specificity. (**A**) Optimization of different staining conditions (temperature and time) via flow cytometry. (**B**) Flow cytometry for other background factors to rule out interference signals. (**C**) Quantification analysis of background factors results (*n* = 3). (**D**) Quantification analysis of staining conditions results (*n* = 3). Data are presented as mean ± SD. Statistical significance was determined using one-way ANOVA with Tukey’s multiple-comparisons test. ns, not significant; **** *p* < 0.0001.

To establish stable application conditions, we evaluated tetramer staining parameters including temperature, incubation time, and buffer composition (Figure 6A). Differences under varying conditions revealed a functional window that maximized TCR tetramer binding while minimizing non-specific interactions. We found that incubation at 0 °C and room temperature for 0.5 h, 1 h, or 2 h failed to accomplish tetramer specific staining. However, incubation at 37 °C for 0.5 h yielded a distinct positive cell population at 60.8% (Figure 6A), with the positive rate increasing with extended incubation time. Crucially, under 37 °C incubation for 2 h, neither streptavidin alone nor alternative buffer components produced detectable background staining (Figure 6B,C). These data support parameters that reproducible tetramer–TCR interactions occur without background interference.

**Figure 7:**
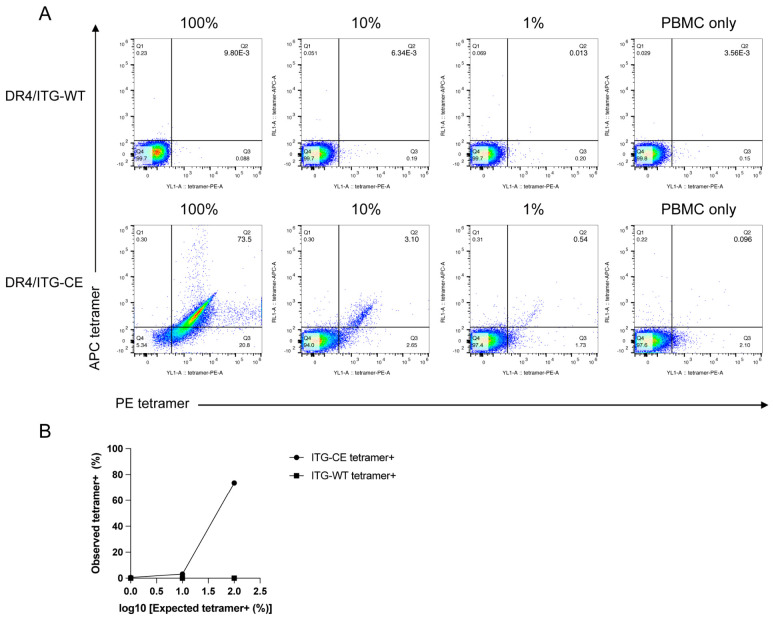
Detection of ITG-CE-specific TCR-transduced CD4^+^ J76 cells in a PBMC background. (**A**) ITG-CE TCR cells were serially diluted into healthy donor PBMCs at frequencies of 100%, 10%, 1%. Mixed cell populations were stained with DR4/ITG-WT tetramer and DR4/ITG-CE tetramer (PE- and APC-conjugated) and analyzed by flow cytometry. Representative flow cytometry plots are shown. (**B**) Quantification analysis of DR4/ITG-CE tetramer sensitivity (*n* = 1).

To further evaluate the ability of the optimized tetramer staining protocol to detect rare antigen-specific cells within a heterogeneous cellular environment, ITG-CE TCR cells were serially diluted into healthy donor PBMCs at defined frequencies (100%, 10%, 1%, and 0%), and tetramer staining was performed using the optimized protocol. As shown (Figure 7A), distinct tetramer-positive populations remained detectable across all dilution levels and exhibited a progressive decrease corresponding to the reduction in ITG-CE TCR cell frequency, whereas PBMC-only controls displayed minimal background staining. Notably, DR4/ITG-WT tetramer staining yielded results comparable to the PBMC-only control across all dilutions, consistent with the lack of TCR recognition of the wild-type epitope and confirming the specificity of the DR4/ITG-CE tetramer. Quantitative analysis revealed a strong correlation between the expected frequency of ITG-CE TCR cells and the observed frequency of tetramer-positive events (Figure 7B), further confirming the quantitative detection capability of the tetramer within heterogeneous cellular samples. Collectively, these findings demonstrate that the DR4/ITG-CE tetramer specifically identify antigen-reactive cells against a complex PBMC background while retaining sensitivity for detecting low-frequency target populations.

## 3. Discussion

This study advances the technology by extending the MHC Class II tetramer system to the emerging field of PTM neoantigens [48]. Traditional designs typically emphasize classical epitopes while overlooking the biochemical diversity of antigenic peptides created by PTMs. In this study, a comprehensive HLA-DR4 platform specifically designed to detect PTM autoimmune neoantigens was established. By focusing on the carboxyethyl-modified ITG-CE epitope, we address a gap in the tooling of how to validate the affinity of structurally altered autoantigens for MHC Class II alleles in vitro and how to utilize CD4^+^ TCR cell lines to distinguish this PTM-dependent structural change.

This study provided a high-throughput neoantigen screening platform that is readily accessible and facilitates verification of batch-to-batch stability. Parameters influencing fluorescence behavior and interaction patterns, such as background fluorescence from buffer ingredients and volume, are systematically evaluated to support reproducible antigen peptide affinity ranking. The intermediate peptide was designed to obtain fluorescently labeled FAM-pep/DR intermediates, enabling tracking of the entire high-affinity peptide exchange process in real time, avoiding the step of fluorescence labeling of candidate peptides one by one in traditional FP experiments. This strategy reduces operational complexity, saving experimental cycles while enhancing throughput and lowering costs [49]. The optimized FP technology is applicable for high-throughput peptide screening and affinity assessment of MHC Class II peptides. By integrating peptide competition results with subsequent tetramer flow cytometry data, it facilitates in-depth analysis of antigen peptide MHC restriction and immunogenicity. Furthermore, it provides a new quantitative perspective on HLA-DR4 interactions with neoantigens, bridging PTM peptides with MHC Class II affinity pairing and AUIDs behavior.

Purified monomers were assessed for biotinylation efficiency to ensure compatibility with streptavidin-mediated tetramerization. Removal of the placeholder CLIP peptide enabled HLA-DR4 to bind the ITG-CE neoantigen, with the structural homogeneity of the prepared monomers supporting transition to functional tetramer. Functional evaluation of the DR4/ITG-CE tetramer corroborated successful PTM peptide loading, with structural integrity confirmed by molecular analysis. PTM-loaded tetramers exhibited strong positive staining on specific TCR cells whilst remaining unstained on irrelevant controls, suggesting PTM-dependent specific recognition of target TCRs by the tetramer.

Together, these enhancements collectively constitute a coherent and reproducible MHC tetramer platform that surpasses traditional tetramer workflows, encompassing monomer protein design, quantitative peptide evaluation, tetramer assembly, and TCR based functional validation. It demonstrates high specificity for matched PTM neoantigen-specific TCRs while maintaining minimal background staining. We confirm that PTM epitopes generate distinct and high-fidelity TCR recognition signals, providing a modular and scalable framework for studying PTM driven autoimmune antigens—including Citrullination, Acetylation, Phosphorylated, Glycosylated, Oxidative Modification, and others—beyond carboxyethyl modification [50,51,52,53].

This platform facilitates the generation of structurally correct pMHC molecules bearing PTM neoantigens, enabling the preparation of functional HLA-DR tetramers. It can be conveniently applied to a wide range of PTM neoantigens associated with AUIDs, cancer immunity, and chronic inflammation [31,54]. Concurrently, it offers opportunities to track neoantigen-specific CD4^+^ T cells in clinical samples and discover autoreactive TCRs, potentially providing novel diagnostic markers or biomarkers of disease activity [55]. This methodology can be extrapolated to additional HLA alleles (e.g., the ongoing work on DR15) [56], supporting potentially investigations into the genetic basis of disease susceptibility. Such advancements enable the elucidation of co-immune mechanisms underlying HLA-associated autoimmunity, providing pivotal tools for developing therapeutic strategies such as blocking antibodies and vaccines [52,57].

## 4. Materials and Methods

### 4.1. HLA-DR4 Molecular Cloning of Protein Expression Constructs

The HLA-DR4 and HLA-DM complexes were synthesized according to the method described in prior research [58], and the codon-optimized genes of interest were inserted into the pFastBac vector (Tsingke Biotech, Beijing, China). The construct was engineered based on a previous description [59].

Specifically, for the human HLA-DR4 β-chain construct (DRB1*04:02, IMGT/HLA Acc No. HLA00687), the molecule consists of a hydrophobic signal sequence at the N-terminal of the β chain, a Strep Tag sequence (WSHPQFEK), a Class II-associated invariant chain peptide (CLIP_383–391_) sequence (PVSKMRMATPLLMQA), a (GAGSGGGGS) linker bearing a thrombin cleavage site (LVPRGS), the mature polypeptide sequence β_1–198_ lacking the intracellular domain, and a second linker (GGGS)3 containing a 3C cleavage site, immediately followed by the basic leucine zipper dimerization domain [60]. The BirA biotinylation site (GLNDIFEAQKIEWHE) and the Flag Tag (DYKDDDDK) are connected to the C-terminal tail via a spacer (GGS). The human HLA-DR4 α-chain construct (DRA*01:01, IMGT/HLA Acc No. HLA00662) is similarly composed of the mature polypeptide sequence α_1–191_ lacking the intracellular domain and with signal peptide, a (GGGS)3 linker containing a 3C cleavage site, and an acidic leucine zipper dimerization domain. The His Tag (HHHHHHHH) is connected to the C-terminus via a spacer (GGS). The amino acid sequence of HLA-DR4 α-chain and β-chain is shown in Table 1.

HLA-DM chaperonin design and construction [42]: For the human HLA-DM β-chain construct (DMB*01:01, IMGT/HLA Acc No. HLA00489), the molecule comprises the mature polypeptide sequence β_1–200_ lacking the intracellular domain and with signal peptide. In this construct, the glycosylation site was mutated (β N92D), and a Flag Tag was connected to the C-terminus via a spacer (GGS). For the human HLA-DM α-chain construct (DMA*01:01, IMGT/HLA Acc No. HLA00485), the molecule comprises the mature polypeptide sequence α_1–204_ lacking the intracellular segment and with signal peptide. Similarly, the specific glycosylation site was mutated (α N165D), and a Strep Tag was fused to the C-terminus via a linker (GGGS). These site-specific mutations (DMα N165D and DMβ N92D) were strategically introduced to reduce glycosylation heterogeneity, maintain in vitro expression stability, and ultimately improve tetramer assembly efficiency.

### 4.2. Antibodies and Antigen Peptides

The FAM-conjugated EBNA1_482–496_ peptide (FAM-AEG, AEGLRALLARSHVER) (Sangon Biotech, Shanghai, China) was dissolved in 10% acetonitrile and 90% H_2_O. Positive peptide influenza HA_307–319_ (PC, PKYVKQNTLKLAT), wild-type ITGA2B peptide (ITG-WT, FLCPWRAEGGQCPS) (Sangon Biotech, Shanghai, China), and the neoantigen peptide (ITG-CE, FLCPWRAEGGQCcePS) (Genscript, Nanjing, China) were dissolved in DMSO to prepare a 10 mM stock solution, which was subsequently diluted with ddH_2_O to form the working solution. All peptide sequences were represented by standard single-letter codes, with purity ≥ 95%. Peptide affinity was predicted using netMHCpan 4.1.

The monoclonal antibody of ITG-CE (anti-CE) recognizes carboxyethyl modification sites, generously provided by Dr. Zhai. Additional reagents include anti-Strep II Tag Antibody (Cat. AF2927, Beyotime, Shanghai, China), anti-Biotin antibody (Cat. A0305, Beyotime, Shanghai, China), anti-His Tag Antibody (Cat 652504, BioLegend, Santa Clara, CA, USA), anti-Flag Tag Antibody (Cat. A02364-50, Genscript, Nanjing, China).

### 4.3. Cells Culture for Transient Production of HLA-DR4 Protein Complexes

The acquired ExpiSf9 cells (Cat. A39111, Thermo Fisher Scientific, Waltham, MA, USA) were proliferated and transfected following the manufacturer’s instructions. Briefly, 800 uL of Enhancer solution was supplemented into a 1 L flask containing 200 mL of Expi293 cells suspension (cell density 5 × 10^6^ cells/mL), and the mixture was incubated overnight at 27 °C under continuous shaking at 125 rpm. After 20 h, cells were infected with a high-titer virus stock (≥1 × 10^8^ ivp/mL) at a multiplicity of infection (MOI) of 5. The flask was placed on the shaker using the conditions described above and incubated for a total of 4 days from the start of transfection. The supernatant was collected from the transfection solution.

### 4.4. Purification of Native HLA-DR4 Complexes

The supernatant was collected and pre-cleared by 3000 × *g* cryo-centrifugation for 30 min [61], followed by passage through a 0.22 μm filter (Cat. 342101, NEST, Wuxi, China). Subsequently, the resulting filtrate was concentrated with a 10 kDa molecular weight cutoff (MWCO) ultrafiltration unit (Amicon Ultra-15, Cat. UFC9010, Millipore, Burlington, MA, USA) and exchanged into Tris-buffered saline (TBS, 10 mM Tris, 150 mM NaCl, pH 8.0). Purification was performed on Anti-Flag Tag Affinity Resin (Cat. L00432, GenScript, Nanjing, China). Binding proteins were washed with 10 column volumes of equilibration buffer (50 mM Tris-HCl, 150 mM NaCl, pH 7.4) and eluted with elution buffer (3–4 M NaCl, pH 7.4). The eluate was then buffer-exchanged into Tris-buffer (10 mM Tris-HCl, pH 8) with a new 10 kDa ultrafiltration unit for secondary concentration. Finally, protein concentration was determined by absorbance measurement at A280 following the method previously described on NanoDrop 2000 spectrophotometer (Thermo Fisher Scientific, Waltham, MA, USA) [40].

### 4.5. Native Gel Electrophoresis-Based Characterization of Peptide Binding to HLA-DR4 Complexes

MHC monomer or complexes samples were mixed in a 5:1 ratio with SDS-PAGE protein loading buffer (Cat. P0286, Beyotime, Shanghai, China) containing 2-mercaptoethanol, and the mixture was denatured at 100 °C for 10 min. Alternatively, samples were directly mixed with non-denaturing non-reducing protein loading buffer (Cat. P0016N, Shanghai, China) lacking 2-mercaptoethanol at a 5:1 ratio. Subsequently, the samples were loaded onto 4–20% polyacrylamide gel (Cat. M00656, Genscript, Nanjing, China) for 2 h at 110 V. Gel imaging after Coomassie Brilliant Blue staining (Cat. P0003S, Beyotime, Shanghai, China) was performed using the NuGenius Gel Documentation System (Syngene, a division of Synoptics Ltd., Cambridge, Cambridgeshire, UK). Western blot images were acquired using an e-BLOT Touch Imager gel imaging system (Shanghai e-Blot Photoelectric Technology Co., Ltd., Shanghai, China). Data analysis was performed using ImageJ (version 1.54).

### 4.6. Fluorescence Polarization Assay for Detecting Peptide Exchange

Thrombin was added prior to the experiment. And FAM-AEG (500 nM), where the N-terminus of the peptide attached with lysine was modified to cysteine for conjugation with the FAM maleimide derivative, was incubated with HLA-DR4 (1 μM) and HLA-DM (500 nM) for 6 h in sodium citrate buffe (SCB, 150 mM sodium chloride with 50 mM sodium citrate buffer, pH 5.2) at 37 °C to generate a fluorescent FAM-AEG loaded HLA-DR4 pMHC (FAM-AEG/DR4) intermediate complexes, which was then purified via Ni-NTA affinity chromatography column (Cat. C600332, Sangon Biotech, Shanghai, China). Competitive binding capacity of candidate peptide was performed in a 100 μL volume all-black 96-well flat-bottom plate (Cat. FCP966, Beyotime, Shanghai, China). Preliminary screening of candidate peptides for competitive binding capacity was performed using FAM-AEG/DR4 (100 nM) and DM (25 nM) in SCB. As previously described [36], FP measurements were performed at 25 °C employing a Synergy H1 full-featured microplate reader (Biotek Instruments, a part of Agilent Technologies, Winooski, VT, USA) configured with a Green FP filter set (Ex 485/20 nm, Em 528/20 nm, Cat. 8040561, Agilent BioTek, Winooski, VT, USA). The 2:1 molar ratio of HLA-DR4 to HLA-DM in the intermediate complex formation was optimized to ensure adequate saturation of the fluorescent tracer while providing sufficient catalytic exchange activity.

### 4.7. Peptide-Loaded HLA-DR4 Tetramer Formation

MHC Class II molecules were biotinylated according to the manufacturer’s protocol. Briefly, 0.25 µg of BirA enzyme (Cat. BI001, iGenebio, Guangzhou, China) was added per 1 nmol of HLA-DR4 protein, incubated at 30 °C for 30–40 min. After biotinylation was completed, the monomers were transferred to a 10 kDa MWCO ultrafiltration unit (Amicon Ultra-0.5, Cat. UFC5010, Millipore, Burlington, MA, USA) and washed with TBS to remove excess biotin.

Subsequently, the CLIP peptide and linker were cleaved with thrombin (Cat. T9326, MilliporeSigma, Burlington, MA, USA) [49]. A total of 1 nmol of HLA-DR4 protein was incubated with 0.5 μg NIH thrombin at 37 °C for 1 h. The efficiency of thrombin was confirmed by SDS-PAGE analysis based on changes in the molecular weight of the HLA-DR4 β chain. Subsequently, the system was incubated in SCB for 16 h with the addition of chaperonin DM and the target peptide (molar ratio of HLA-DR4:HLA-DM:peptide = 4:1:100). This non-equimolar ratio was implemented because HLA-DM functions as a catalytic enzyme rather than a stoichiometric component, consistently facilitating efficient placeholder displacement without being consumed, as supported by established protocols [51,62]. The exchanged pMHC was transferred to His Tag resin (Cat. L00436, Genscript, Nanjing, China) for purification and resalted to PBS (Cat. C3580-0500, Vivacell, Shanghai, China) buffer using a new 10 kDa ultrafiltration unit. For long-term storage, pMHC was resuspended with 15% (*v*/*v*) glycerol before being stored at −80 °C.

pMHC was performed with fluorescently labeled streptavidin (SA) tetramerization at a 4:1 molar ratio by adding PE-streptavidin (Cat. 405203, BioLegend, Santa Clara, CA, USA) or APC-streptavidin (Cat. 405207, BioLegend, Santa Clara, CA, USA) in five batches over 1 h on ice. The final tetramer concentration was 2 μM (concentration relates to the HLA-DR4 protein only component of the tetramer). Residual-free streptavidin sites were blocked by supplementation with excess unlabeled biotin, followed by overnight incubation at 4 °C. Upon assembly completion, up to 2 × 10^6^ Jurkat 76 (J76) cells (detailed in Section 4.8) were stained using 20 nM tetramer (concentration relates to the HLA-DR4 monomer only component of the tetramer). The tetramer was stored at 4 °C prior to staining.

### 4.8. Cells Preparation and Tetramer Staining for Flow Cytometry

For cell lines staining, we used Jurkat-derived TCR-knocked Jurkat 76 (J76) cell lines that stably express human-CD4 (Figure 4D) [63]. These TCRα/β^–/–^ and CD4^+^ J76 cell lines (Shanghai Yiling Pharmaceutical Co., Ltd., Shanghai, China) were engineered to express either neoantigen receptor ITG-CE TCR or, as comparators, TCRs specific for CMV pp65_495–503_ (NLVPMVATV) and FLU M1_58–66_ (GILGFVFTL). The procedure was as follows: DR4/ITG-WT tetramer (loaded ITG-WT peptide), DR4/ITG-CE tetramer (loaded ITG-CE peptide), or DR4/CLIP (un-exchanged peptide) tetramer were refolded, and 1 × 10^6^ cells were stained at 37 °C for 1 h. Prior to flow cytometry harvesting, cells were subjected to two sequential washes, washed twice with fluorescence activated cell sorting (FACS) buffer containing 2% (*w*/*v*) bovine serum albumin (BSA, Cat. abs9157-100g, Absin, Shanghai, China) and 5 mM EDTA (Cat. AM9260G, Thermo Fisher Scientific, Waltham, MA, USA).

For Spike-in tetramer staining in PBMC, the healthy donor PBMCs (Lot No. P123060411C-B, Milecell Bio, Shanghai, China) were used as background cells. Cryopreserved PBMCs were rapidly thawed in a 37 °C water bath and transferred into 10 mL pre-warmed RPMI-1640 complete medium (supplemented with 10% FBS). Cells were centrifuged at 500× *g* for 5 min, resuspended in complete medium, and counted before use. ITG-CE TCR cells were mixed with PBMCs at defined frequencies (100%, 10%, 1%, and 0%). The total cell number for each sample was adjusted to 1 × 10^6^ cells. Cell mixtures were stained with PE- and APC-conjugated tetramers at 37 °C for 1 h in the dark. Samples were gently mixed every 15 min during incubation. After tetramer staining, cells were incubated with LIVE/DEA Fixable Green Dead Cell Stain Kit (Cat. L34966, Thermo Fisher Scientific, Waltham, MA, USA) for 15 min at 4 °C. Cells were subsequently washed twice with FACS buffer at 500× *g* for 5 min and resuspended in 200 μL PBS for acquisition.

Cells were acquired using either the Attune Nxt instrument (Thermo Fisher Scientific, Waltham, MA, USA) or BD FACSCelesta Multicolor Flow Cytometer (BD Biosciences, San Jose, CA, USA). Data analysis was performed using FlowJo (version 10.9.0).

### 4.9. Natively Paired TCRα/β Library Cloning

The pCDH vector (Cat. CD526A-1, System Biosciences, Palo Alto, CA, USA) was engineered to achieve surface expression of TCRs [64]. After digestion by restriction enzymes, the vector and amplified TCR fragment were ligated using HiFi DNA Assembly Master Mix (Cat. E2621, New England Biolabs, Ipswich, MA, USA). The assembled product, which is approximately 8.94 kbp in size, was recovered through column-based purification employing the PCR & DNA Cleanup Kit (NEB, Cat. T1030, New England Biolabs, Ipswich, MA, USA) and transformed into stbl3 competent cells (Cat. B528420, Sangon Biotech, Shanghai, China). Plasmids containing TCR libraries were purified using the NucleoBond Xtra Midi Kit (Cat. 740410.50, MACHEREY-NAGEL, Düren, Germany) for transfection-grade plasmid DNA.

### 4.10. Cell Transfection and Viral Concentration

293T adherent cells were cultured in DMEM medium (Cat. C11995500BT, Gibco, Thermo Fisher Scientific, Waltham, MA, USA) supplemented with 10% fetal bovine serum (FBS, Cat. SV30208.02, HyClone, Logan, UT, USA) at 37 °C in a 5% CO_2_ incubator. For lentiviral packaging, cells were seeded in 6-well plates and cultured to 70% confluency in antibiotic-free complete medium prior to transfection. All plasmids were settled to 1 µg/µL; a mixture was prepared comprising 100 µL Opti-MEM Reduced Serum Medium (Cat. 31985070, Gibco, Thermo Fisher Scientific, Waltham, MA, USA), 1.5 μg PSPAX (Addgene plasmid #12260, Addgene, Inc., Watertown, MA, USA), 0.5 μg PMD2G plasmid (Addgene plasmid #12259, Addgene, Inc., Watertown, MA, USA), and 2 μg lentiviral target plasmid. The mixture was allowed to equilibrate at room temperature for 5 min, after which 12 μL ViaFect™ Transfection Reagent (Cat. E4981, Promega (Beijing) Biotech, Beijing, China) was added, and the mixture was incubated at room temperature for an additional 10 min. The final mixture was added dropwise onto 293T cells cultured in 6-well plates and incubate at 37 °C for 2 days. Viral supernatants were harvested from transfected 293T cells and clarified by centrifugation at 800× *g* for 10 min. For the subsequent transduction of J76 cells, viral particles in the supernatant were concentrated by combining with Lenti-X Concentrator (Cat. 631232, Takara Bio, Kusatsu, Shiga, Japan) at a 3:1 (*v*/*v*) ratio, followed by overnight incubation at 4 °C and virus pellet collection.

### 4.11. TCR Transduction

TCR maps were generously provided by Dr. Zhai. As detailed in Section 4.9, the constructs encoding TCRβ and TCRα were successfully cloned into the pCDH lentiviral vectors. The concentrated viral suspension (harvested from one well of a 6-well plate) was centrifuged at 1500 × *g* for 45 min at 4 °C; the pellet was resuspended in 50 µL DMEM medium (Cat. C11995500BT, Gibco, Thermo Fisher Scientific, Waltham, MA, USA) containing 8 μg/mL Polybrenes (Cat. H9268, MilliporeSigma, Burlington, MA, USA) and 10 mM HEPES (Cat. 15630-080, Gibco, Thermo Fisher Scientific, Waltham, MA, USA). A total of 1 × 10^5^ J76 cells were placed in a well of a 96-well plate, followed by the addition of the 50 µL viral pellet suspension. The cells were transduced by centrifugation at 2400 rpm for 2–3 h at 32 °C; then, cells were resuspended in 200 µL RPMI 1640 medium (Cat. C11875500BT, Gibco, Thermo Fisher Scientific, Waltham, MA, USA) supplemented with 10% FBS and were incubated at 37 °C in a 5% CO_2_ environment. On the following day, the cells were transferred to a 48-well plate for continued culture, observation, and routine maintenance. Cells successfully expressing the target TCR were subjected to monoclonal sorting via BD FACSMelodyTM Cell Sorter (BD Biosciences, San Jose, CA, USA). Data analysis was performed using FlowJo (version 10.9.0).

## 5. Conclusions

In summary, this study establishes a robust and scalable HLA-DR4-based MHC Class II tetramer platform for the detection and validation of PTM neoantigens. By integrating an optimized fluorescence polarization assay with a FAM-labeled intermediate peptide strategy, the platform improves peptide exchange efficiency while reducing experimental cost and complexity. Using the carboxyethyl-modified ITG-CE epitope as a model, we demonstrated sensitive and specific detection of PTM-dependent TCR recognition and CD4^+^ T cell autoreactivity. The modular workflow is readily adaptable to other PTM types and HLA alleles, providing a versatile framework for studying PTM neoantigen presentation, immune monitoring, and the mechanisms underlying HLA-associated autoimmune diseases.

## Figures and Tables

**Table 1:** The amino acid sequence of HLA-DR4.

Name	Sequence (Amino Acids)
HLA-DR4 α	MAISGVPVLGFFIIAVLMSAQESWAIKEEHVIIQAEFYLNPDQSGEFMFDFDGDEIFHVDMAKKETVWRLEEFGRFASFEAQGALANIAVDKANLEIMTKRSNYTPITNVPPEVTVLTNSPVELREPNVLICFIDKFTPPVVNVTWLRNGKPVTTGVSETVFLPREDHLFRKFHYLPFLPSTEDVYDCRVEHWGLDEPLLKHWEFDAPSPLPETTEGGGSGGGSCSRGGLEVLFQGPKFGGSTTAPSAQLEKELQALEKENAQLEWELQALEKELAQGGSHHHHHHHH *
HLA-DR4 β	MVCLKFPGGSCMAALTVTLMVLSSPLALAGSWSHPQFEKGSPVSKMRMATPLLMQAGAGSLVPRGSGGGGSGDTRPRFLEQVKHECHFFNGTERVRFLDRYFYHQEEYVRFDSDVGEYRAVTELGRPDAEYWNSQKDILEDERAAVDTYCRHNYGVVESFTVQRRVYPEVTVYPAKTQPLQHHNLLVCSVNGFYPGSIEVRWFRNGQEEKTGVVSTGLIQNGDWTFQTLVMLETVPRSGEVYTCQVEHPSLTSPLTVEWRARSESAQSKGGGSGGGSCGGGSLEVLFQGPKFGGSTTAPSAQLKKKLQALKKKNAQLKWKLQALKKKLAQGSDYKDDDDKGSGLNDIFEAQKIEWHE *

* This represents a stop codon.

## Data Availability

The original data supporting the conclusions of this study are included within the article. Additional data related to this study are available from the corresponding author upon reasonable request.

## Abbreviations

The following abbreviations are used in this manuscript:

AUIDs	autoimmune diseases
MHC	Major Histocompatibility Complex
RA	rheumatoid arthritis
T1D	type 1 diabetes
IBD	inflammatory bowel disease
SLE	systemic lupus erythematosus
HLA	human leukocyte antigens
AS	Ankylosing Spondylitis
MS	multiple sclerosis
pMHC	peptide-MHCPTM, post-translational modification
HLA-DR4	HLA-DRB1*04:02 and HLA-DRA*01:01
TCR	T cell receptor
FP	fluorescence polarization
CLIP	class II-associated invariant chain peptide
FAM-AEG	FAM-conjugated EBNA1482-496 peptide
PC	positive peptide
ITG-WT	wild type ITGA2B peptide
ITG-CE	carboxyethyl-modified neoantigen ITGA2B peptide
anti-CE	antibody of ITG-CE
MWCO	molecular weight cutoff
TBS	tris-buffered saline
SCB	sodium citrate buffe
FAM-AEG/DR4	FAM-AEG loaded HLA-DR4 pMHC
SA	streptavidin
J76	TCR-knocked Jurkat 76 cell lines
DR4/ITG-WT	HLA-DR4 tetramer loaded ITG-WT peptide
DR4/ITG-CE	HLA-DR4 tetramer loaded ITG-CE peptide
DR4/CLIP	un-exchanged peptide HLA-DR4 tetramer
FACS	fluorescence activated cell sorting
BSA	bovine serum albumin
IMGT	International ImMunoGeneTics information system
SEC-MALS	Size Exclusion Chromatography with Multi-Angle Light Scattering
SPR	surface plasmon resonance
BLI	Bio-Layer Interferometry
DR4	HLA-DR4 monomer linked CLIP
PC/DR4	PC peptide loaded HLA-DR4 pMHC
WT/DR4	ITG-WT peptide loaded HLA-DR4 pMHC
CE/DR4	ITG-CE peptide loaded HLA-DR4 pMHC
ITG-CE TCR cells	ITG-CE-specific TCR-transduced CD4^+^ J76 cells
